# Comparison of the prophylactic antithrombotic effect of indobufen and warfarin in patients with nephrotic syndrome: a randomized controlled trial

**DOI:** 10.1080/0886022X.2022.2163505

**Published:** 2023-01-13

**Authors:** Xin-Yi Gao, Yue-Ming Liu, Dan-Na Zheng, Yi-Wen Li, Hua Li, Xiao-Ling Xiong, Hong-Yu Chen, Hua Wang, Xiao-Yong Yu, Kai Qu, Juan Jin, Bo Lin, Qiang He

**Affiliations:** a Urology & Nephrology Center, Department of Nephrology, Zhejiang Provincial People’s Hospital, Affiliated People’s Hospital, Hangzhou Medical College, Hangzhou, China; b Department of Nephrology, Sir Run Shaw Hospital, Zhejiang University School of Medicine, Hangzhou, China; c Department of Nephrology, Hangzhou Hospital of Traditional Chinese Medicine, Hangzhou, China; d Department of Nephrology, Shaanxi Traditional Chinese Medicine Hospital, Xi’an, China; e Department of Nephrology, First Affiliated Hospital of Zhejiang Chinese Medical University (Zhejiang Provincial Hospital of Traditional Chinese Medicine), Hangzhou, China

**Keywords:** Antithrombotic effect, indobufen, warfarin, nephrotic syndrome, randomized controlled trial

## Abstract

**Purpose:**

The risk of thromboembolic events is elevated in patients with nephrotic syndrome, and warfarin use has been associated with an increased risk of bleeding. Indobufen, a selective cyclooxygenase-1 inhibitor, is currently being evaluated for the prevention of thromboembolic events in nephrotic syndrome. This study aimed to compare the efficacy and safety of indobufen with that of warfarin in patients with nephrotic syndrome.

**Materials and methods:**

This multicenter, randomized, three-arm, open-label, parallel controlled trial involved a total of 180 adult patients with nephrotic syndrome from four centers in China. Patients were randomly assigned to receive 100 mg indobufen (bid), 200 mg indobufen (bid), and 3 mg warfarin (qd) daily for 12 weeks. The primary endpoints included thromboembolic and bleeding events, while laboratory results and adverse events constituted secondary endpoints.

**Results:**

No thromboembolic events occurred in the high-/low-dose indobufen and warfarin groups. Moreover, the use of a low dose of indobufen significantly reduced the risk of minor bleeding events compared with warfarin use (2% versus 18%, *p* < .05). Finally, adverse events were more frequent in warfarin-treated patients.

**Conclusions:**

This study found that indobufen therapy provided equivalent effects in preventing thromboembolic events compared with warfarin therapy, while low dose of indobufen was associated with a reduced risk of bleeding events, thus it should be recommended for the prevention of thromboembolic events in clinical practice in patients with nephrotic syndrome.

**Trial registration number:**

ChiCTR-IPR-17013428.

## Introduction

Nephrotic syndrome is characterized by a urine protein loss > 3.5 g/24 h, accompanied by hypoalbuminemia and edema, with an annual incidence of 1–3 per 100,000 adults [1]. Nephrotic syndrome has been associated with an increased risk of thromboembolic events, predominantly occurring in the veins, including deep venous thrombosis, renal venous thrombosis, and pulmonary embolism, with prevalence of 15%, 27.9%, and 12.8%, respectively [2]. Platelet activity and cohesion aggregation play important roles in the risk of thromboembolic events in patients with nephrotic syndrome. In patients with nephrotic syndrome, thrombocytosis potentially causes a decrease in red blood cell variability [3] and an increase in the von Willebrand factor level, which potentially induces platelet aggregation to the vascular wall and increases platelet adhesion [4]. In addition, platelet aggregation is increased in patients with nephrotic syndrome *in vitro* [5].

Currently, warfarin is widely used as a prophylactic anticoagulant; however, its risk of bleeding events is significantly high [6,7]. Indobufen, a selective and reversible cyclooxygenase-1 inhibitor, effectively inhibits platelet activation, adhesion, and aggregation [8]. Studies have demonstrated that indobufen reduces platelet factors 3 and 4, thereby reducing the activation of coagulation factors II and X, which play important roles in blood coagulation [9]. Moreover, the use of indobufen versus placebo showed reduced risk of ischemic events in patients with heart disease, and indobufen versus acetylsalicylic acid plus dipyridamole was associated with the higher one-year cumulative patency rate for patients after femoropopliteal bypass [10,11]. A prior study found that thrombotic recanalization or disappearance occurred in patients with deep venous thrombosis treated with indobufen 600 mg/day, thus suggesting that indobufen use potentially reduces the risk of deep venous thrombosis [12]. Our previous study on a rat model of adenine-induced chronic kidney disease revealed that indobufen was associated with lower degree of inflammatory infiltration and fibrosis of the renal tissue compared with warfarin [13]. Moreover, indobufen may be associated with lower prothrombin time (PT) and activated partial thromboplastin time (APTT) levels. However, although indobufen may be useful for thromboembolic events in patients with nephrotic syndrome, no randomized controlled trial has investigated the efficacy and safety outcomes of indobufen in patients with nephrotic syndrome. Therefore, we performed this study to compare the efficacy and safety of indobufen with that of warfarin in patients with nephrotic syndrome.

## Methods

### Trial design and oversight

This prospective, multicenter, randomized clinical trial was conducted at four centers in China. All patients provided written informed consent before participation, and the institutional review board of each center approved the protocol before the initiation of the study. The coauthors contributed to the first draft of the original manuscript and revision of the original manuscript. This study was registered at http://www.chictr.org.cn (ChiCTR-IPR-17013428), and the current trial was performed following the consolidated standards of reporting trial CONSORT guidelines [14].

### Trial population

Adult patients with nephrotic syndrome were eligible for inclusion in this study, and the inclusion criteria were as follows: (1) age between 18 and 75 years; (2) 24-h urinary protein excretion > 3.5 g and/or serum albumin < 30 g/L; and (3) exposure to thrombosis risk factors, such as recent abdominal surgery, prolonged immobilization, heart failure, and body mass index (BMI) > 35 kg/m^2^. Patients were excluded if they had (1) a history of allergy to indobufen or warfarin and (2) evidence or suspicion of thrombosis at enrollment.

### Randomization and treatment protocol

After enrollment, patients were randomly assigned to three groups in a 1:1:1 ratio, and an observer-blinded approach was followed. Randomization was performed using a random number table with a central computerized system. Group A was administered an oral dose of 100 mg of indobufen twice daily. Group B was administered an oral dose of 200 mg of indobufen twice daily. Group C received 3 mg of warfarin orally once a day. Dose adjustment was based on the PT. The international normalized ratio (INR) was maintained between 1.5 and 3.0.

Patients were followed-up for 12 weeks, and study visits occurred at baseline and at weeks 1, 4, 8, and 12. Patients or researchers had the option of discontinuing treatment early because of unacceptable serious adverse events (SAEs), any change in the patient’s condition that justified discontinuation (decided by the researcher; included the requirement for any drug that treats thrombosis, which was an exclusion criterion), withdrawal of consent (decided by the patient), and complete remission of nephrotic syndrome (24-h urinary protein < 0.3 g, serum albumin > 35 g/L).

### Collected variables

Baseline data included age, sex, weight, BMI, and pathological diagnosis from kidney biopsy samples. BMI is the weight in kilograms divided by the square of the height in meters. Baseline laboratory data obtained from the central laboratory included the following: hemoglobin (Hb), platelet count, serum albumin (Salb), blood urea nitrogen, serum creatinine, APTT, PT, thrombin time (TT), fibrinogen (FIB), and D-dimer. All laboratory examinations were performed at the central hospital laboratory.

### Endpoints

The primary outcomes were the incidence rates of thrombosis and bleeding. Thromboembolic events were confirmed by ultrasound (for deep vein thrombosis), renal magnetic resonance venography (for renal vein thrombosis), or computed tomography pulmonary ventilation-perfusion scan (for pulmonary embolism). Major bleeding referred to any of the following: fatal bleeding, two symptomatic bleeding events at a critical site, bleeding causing the Hb level to drop by ≥ 20 g/L (1.24 mmol/L), or bleeding requiring the transfusion of two or more units of whole blood or red blood cells [15]. Minor bleeding events were defined as bleeding not meeting the criteria for major bleeding but associated with medical intervention; contact with a physician; interruption of the study drug; or discomfort/impairment in performing daily activities, including gastrointestinal bleeding, nasal bleeding, subcutaneous hemorrhage, fundus bleeding, and non-glomerular origin hematuria. The secondary outcome was coagulation function (APTT, PT, TT, and D-dimer). In group C, INR values were also collected. The incidences of AEs and SAEs were recorded from the initial treatment until the final follow-up visit. AEs were summarized using the MedDRA system organ class and the preferred term. An independent data monitoring committee reviewed the cumulative safety data.

### Statistical analysis

Patient demographic, clinical, and laboratory data are described using (1) the mean (±SD) or median (range), depending on the underlying distribution, for continuous variables and (2) frequencies (percentages) for categorical variables. Continuous variables were compared using analysis of variance, while categorical variables were compared using the chi-square test. A mixed linear regression model was applied to assess the potential changes in laboratory data among the groups and various visit time points, and the intercept was random. The reported *p* values were two-sided, and the inspection level was .05. The IBM Statistical Package for the Social Sciences for Windows (version 19.0; IBM, Armonk, NY, USA) was used to perform all statistical analyses.

## Results

### Patients

**Figure 1.**
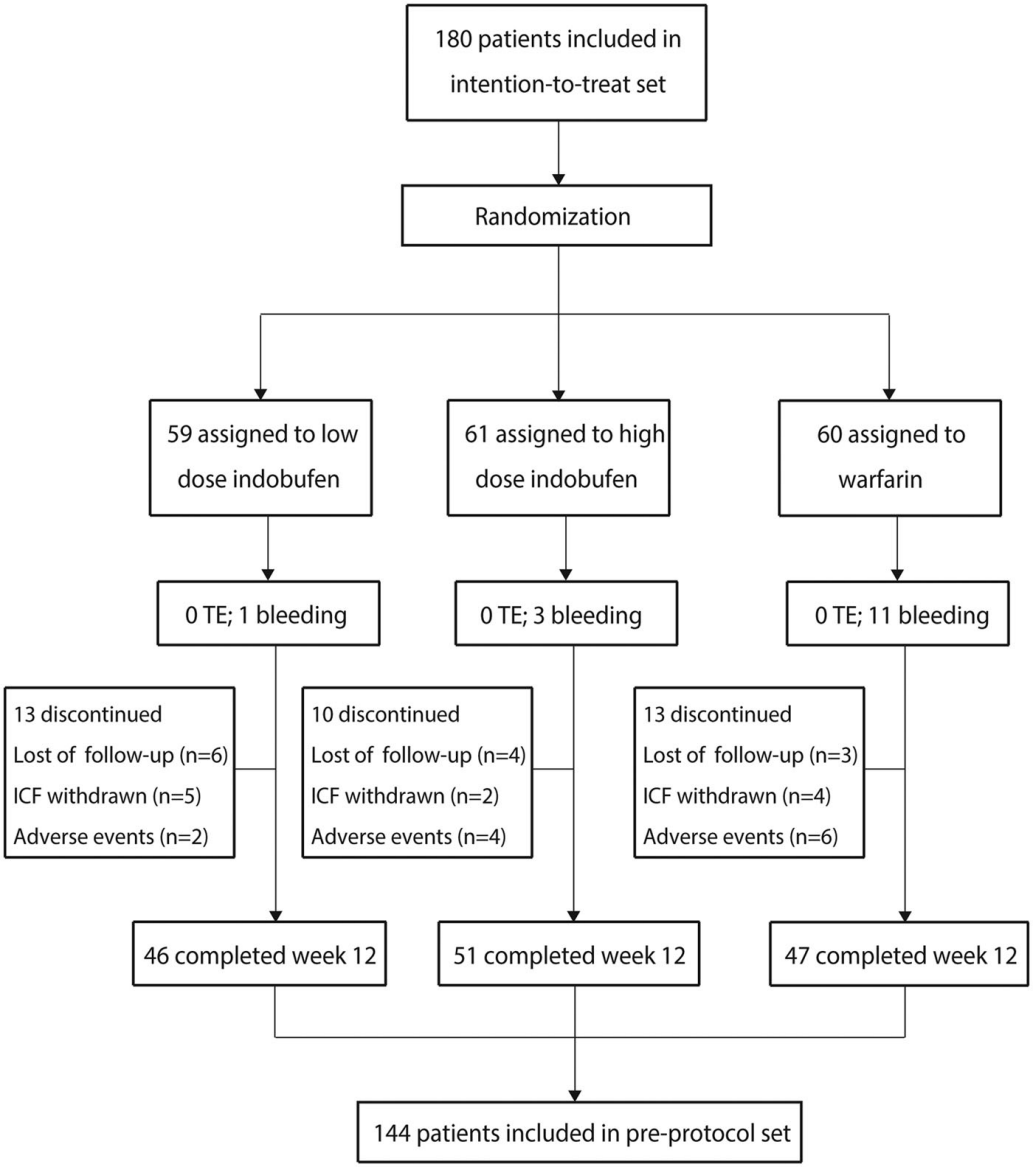
Flow chart for the study population.

Between December 2017 and December 2020, 180 patients from four different centers were randomly assigned (59 in group A, 61 in group B, and 60 in group C) and followed up until February 2021. Nine patients discontinued treatment early during the COVID-19 lockdown. Of the 180 patients, 36 (20%) had missing primary-outcome data. Therefore, the baseline characteristics and primary-outcome analysis were based on the intention-to-treat set, which included 180 patients, while the pre-protocol analysis population included 144 patients (46 in group A, 51 in group B, and 47 in group C) (Figure 1).

### Baseline characteristics

The baseline characteristics of the enrolled patients are shown in Table 1. We noted that the baseline characteristics of the groups were well balanced. The mean adherence was 78% in group A (95% confidence interval [CI]: 67%–89%), 84% in group B (95% CI: 74%–93%), and 78% in group C (95% CI: 68%–89%). No statistically significant differences were noted among the three groups (*p* = .686). Premature discontinuation of the study drug occurred in 13, 10, and 13 patients in groups A, B, and C, respectively.

**Table 1.** Baseline characteristics of enrolled patients.^a^

Variables	Indobufen, 100 mg*2/d (*n* = 59)	Indobufen, 200 mg*2/d (*n* = 61)	Warfarin, INR 1.5–3.0 (*n* = 60)^b^	*p* Value
Age (years, mean ± SD)	47.3 ± 16.7	44.3 ± 15.7	45.5 ± 16.9	.601
Male sex, n (%)	42 (71.2%)	37 (60.7%)	32 (53.3%)	.132
Weight (kg, mean ± SD)	72.1 ± 14.2	69.0 ± 11.4	66.5 ± 14.6	.076
Body-mass index (kg/m^2^)	25.5 ± 4.2	24.9 ± 3.2	24.3 ± 4.0	.215
Pathological type, n (%)				.107
MN	35 (59.3%)	30 (49.2%)	32 (51.7%)	
IgAN	2 (3.4%)	6 (9.8%)	10 (16.7%)	
MCD	8 (13.6%)	8 (13.1%)	11 (18.3%)	
Other	8 (13.6%)	14 (23.0%)	5 (8.3%)	
Unknow	6 (10.2%)	3 (4.9%)	2 (3.3%)	
Laboratory test				
Hb (g/L)	130.70 ± 29.81	134.26 ± 20.71	126.50 ± 25.71	.251
PLT (10^9^/L)	241.07 ± 77.77	244.45 ± 76.02	245.03 ± 61.60	.950
SAlb (g/L)	23.75 ± 7.52	23.26 ± 6.34	23.27 ± 7.11	.911
BUN (mmol/L)	5.4 (4.0, 6.8)	5.4 (4.2, 7.2)	5.9 (4.8, 7.5)	.210
SCr (μmol/L)	75.0 (61.3, 85.4)	73.0 (59.3, 87.2)	67.9 (57.9, 81.6)	.220
APTT (s)	27.66 ± 9.30	27.25 ± 8.63	27.32 ± 10.29	.969
PT (s)	11.5 (10.6, 12.4)	11.1 (10.3, 12.4)	11.6 (10.5, 12.3)	.649
TT (s)	18.12 ± 1.54	18.30 ± 1.41	18.48 ± 2.31	.573
FIB (g/L)	4.6 (3.7, 5.5)	4.2 (3.1, 5.4)	3.9 (3.5, 4.8)	.103
D-dimer (μg/L)	3780 (1325, 4883)	3210 (1710, 4855)	3675 (1727, 4600)	.695
INR	0.89	NA	0.96 ± 0.22	NA

*Note:* APTT: activated partial thromboplastin time; BUN: blood urea nitrogen; FIB: fibrinogen; Hb: hemoglobin; IgAN: Immunoglobulin A nephropathy; MCD: minimal change disease; MN: membranous nephropathy; PLT: platelet; PT: prothrombin time; SAlb: serum albumin; SCr: serum creatinine; TT: thrombin time.

^a^ The analysis of the baseline data was performed in the intention-to-treat set (*n* = 180).

^b^ 3 mg/d at day 1, dose adjustment was based on INR.

### Primary endpoints

The median follow-up time was 10.3 weeks. After 12 weeks of intervention, no thromboembolic and major-bleeding events occurred in the three groups. Minor bleeding occurred in 1 of 59 patients in group A (2%; 95% CI:0%–5%), 3 of 61 patients in group B (5%; 95% CI:0%–11%), and 11 of 60 patients in group C (18%, 95% CI:6%–30%). We noted that patients in group A were associated with a reduced risk of minor bleeding events compared with those in group C (*p* < .05) (Table 2).

**Table 2.** Clinical outcomes (*n* = 180).^a^

Variables	Indobufen, 100 mg*2/d (*n* = 59)	Indobufen, 200 mg*2/d (*n* = 61)	Warfarin, INR 1.5–3.0 (*n* = 60)	*p* Value
Efficacy, *n* (%)				
Thromboembolic events	0	0	0	ns
Safety, *n* (%, 95%CI)^b^				
Bleeding events	1 (2%, 0–5)^c^	3 (5%, 0–11)	11 (18%, 6–30)	.013
Non-glomerular hematuria	0	2	3	
Gastrointestinal bleeding	0	0	1	
Subcutaneous bleeding	1	1	3	
Submucosal hemorrhage	0	0	1	
Subconjunctival bleeding	0	0	1	
Nasal bleeding	0	0	1	
Gum hemorrhage	0	0	1	

^a^ The analysis of efficacy and safety outcomes were performed in the intention-to-treat set (*n* = 180).

^b^ 95% CI of proportions were calculated with the Wilson’s score method. Patients with more than one bleeding event in one same place were only counted once.

^c^ *p* < .05 compared with warfarin group.

### Secondary endpoints

The changes in coagulation function in the groups and at various visits are shown in Table 3. Significant differences in APTT and PT were observed among the groups at weeks 4, 8, and 12 (*p* < .05). Furthermore, the TT level at four weeks among the groups was statistically significant (*p* = .008). The results of the mixed linear regression model are presented in the Supplementary File. First, we noted that the APTT in group C was higher than that in group A (*p* < .001), and the changes in APTT at weeks 4 and 8 were statistically significant. Second, patients in group C had a higher PT than those in group A (*p* < .001), while the changes in PT at weeks 1, 4, 8, and 12 were statistically significant. Third, D-dimer levels at weeks 4, 8, and 12 were significantly reduced. Finally, although the APTT and PT levels among the three groups were similar and within the normal range at baseline, we noted that these levels were reduced in groups A and B and remained high in group C after the intervention.

**Table 3.** Laboratory analysis.

Variables	Timepoints	Indobufen, 100 mg*2/d (*n* = 59)	Indobufen, 200 mg*2/d (*n* = 61)	Warfarin, INR 1.5–3.0 (*n* = 60)	*p* Value
APTT (s)	Baseline	27.66 ± 9.30	27.25 ± 8.63	27.32 ± 10.29	.969
	4 weeks	24.79 ± 7.74	25.10 ± 7.31	35.81 ± 11.15	<.001
	8 weeks	23.45 ± 7.32	22.92 ± 7.11	31.46 ± 10.27	<.001
	12 weeks	25.53 ± 6.56	24.52 ± 7.52	28.99 ± 7.97	.010
PT (s)	Baseline	12.73 ± 4.88	12.62 ± 4.52	13.15 ± 4.80	.813
	4 weeks	12.78 ± 5.69	11.79 ± 4.04	26.72 ± 10.19	<.001
	8 weeks	12.44 ± 5.12	11.94 ± 4.17	21.23 ± 9.85	<.001
	12 weeks	12.48 ± 5.61	11.72 ± 3.93	19.07 ± 7.87	<.001
TT (s)	Baseline	18.12 ± 1.54	18.30 ± 1.41	18.48 ± 2.31	.573
	4 weeks	18.40 ± 1.65	19.03 ± 1.95	17.97 ± 1.58	.008
	8 weeks	17.48 ± 1.45	17.41 ± 1.42	17.46 ± 2.59	.979
	12 weeks	17.62 ± 1.48	19.82 ± 16.85	17.61 ± 2.10	.471
D-dimer (μg/L)	Baseline	3985.18 ± 3340.52	3717.67 ± 3302.78	3439.00 ± 1930.44	.603
	4 weeks	3070.04 ± 2021.52	3481.56 ± 2563.87	3069.02 ± 1879.85	.557
	8 weeks	2929.25 ± 1979.59	3265.00 ± 4105.18	2915.60 ± 2151.31	.783
	12 weeks	2506.67 ± 1795.17	2845.33 ± 2301.67	2610.89 ± 2045.67	.734
INR	Baseline	0.89±…	NA	0.96 ± 0.22	…
	4 weeks	NA	NA	1.87 ± 0.92	…
	8 weeks	0.90±…	NA	1.66 ± 0.72	…
	12 weeks	NA	1.02±…	1.52 ± 0.60	…

*Note:* APTT: activated partial thromboplastin time; PT: prothrombin time; TT: thrombin time.

The AEs in the groups are shown in Table 4. There was one case of cough and another of new-onset tumor in patients treated with a low dose of indobufen, while one case each of cough, hand twitch, nausea, and new-onset tumor was reported among patients treated with a high dose of indobufen. Moreover, one case each of erythra, headache, dry mouth, abdominal distension, diarrhea, and sudden cardiac death was reported among patients treated with warfarin.

**Table 4.** Adverse events (*n* = 180).

Events	Indobufen, 100 mg*2/d (*n* = 59)	Indobufen, 200 mg*2/d (*n* = 61)	Warfarin, INR 1.5–3.0 (*n* = 60)
Erythra	0	0	1
Cough	1	1	0
Headache	0	0	1
Hand twitch	0	1	0
Dry mouth	0	0	1
Abdominal distension	0	0	1
Diarrhea	0	0	1
Nausea	0	1	0
Sudden cardiac death	0	0	1
New onset tumor	1	1	0

Serum albumin was considered an indicator of predisposition for thromboembolic events. At baseline, 35 (76.1%), 41 (80.4%), and 36 (76.6%) patients exhibited serum albumin levels < 28 g/L in groups A, B, and C, respectively. After 12 weeks, the number of patients with serum albumin levels < 28 g/L in groups A, B, and C decreased to 11 (23.9%), 11 (21.6%), and 13 (27.7%), respectively. Moreover, the mean serum albumin levels improved in groups A, B, and C (11.3 [8.9], 11.7, and 10.8 [10.1] g/L), and no significant differences were observed among the three groups (*p* = .678).

At baseline, the INR for patients in group C was 0.93 (0.79–1.01), and it increased to 1.44 (0.95–2.04) and 2.03 (1.44–2.92) on days 3 and 7, respectively. Moreover, the mean INR remained high during follow-up, and a total of 15 patients showed INR > 1.5 after 12 weeks. Furthermore, the mean INRs for patients with and without minor bleeding events were 3.37 (2.11–4.81) and 1.67 (1.22–2.37), respectively, and the difference between patients with and without bleeding events was statistically significant (*p* < .01).

## Discussion

Hypercoagulability, the main pathological change in nephrotic syndrome, is possibly caused by reduced anticoagulants (antithrombin III), increased liver procoagulant synthesis (FIB, factor V, and factor VIII), increased platelet activation and aggregability, and decreased fibrinolytic activity in the kidney [15]. Moreover, the Kidney Disease Improving Global Outcomes clinical practice guidelines indicate that full-dose prophylactic anticoagulants should be administered to patients presenting with hypoproteinemia and high thromboembolism risk [1]. Several prophylactic anticoagulants have already been used in clinical practice, including low-molecular-weight heparin, warfarin, aspirin, and direct oral anticoagulants. However, these prophylactic anticoagulants have several limitations, including poor patient compliance with low-molecular-weight heparin owing to cold storage and intravenous administration [16], the high cost of direct oral anticoagulants [17], various warfarin-related complications [18,19], and aspirin’s limited efficacy [20,21].

The current study was designed as a prospective, multicenter, randomized clinical trial, and it recruited 180 patients with nephrotic syndrome. This study found no thromboembolic events in the three groups, while low doses of indobufen were associated with a effectively reduced risk of minor bleeding events compared with warfarin. Moreover, APTT and PT levels in the warfarin group were higher than those in the low-dose indobufen group. Finally, one death occurred in the warfarin group; the patient died of sudden cardiac arrest between visits 3 and 4. Finally, other nonspecific AEs predominantly occurred in the warfarin group.

This study did not observe any thromboembolic events in the three groups, suggesting that indobufen has an antithrombotic effect equivalent to that of warfarin, and it may be superior to warfarin owing to the significantly lower incidence of minor bleeding events. The trends in serum albumin and creatinine levels in the three groups were similar, suggesting that indobufen did not affect the efficacy of immunosuppressants in the treatment of nephrotic syndrome compared with warfarin. Moreover, the mean APTT and PT values were higher than normal (APTT:23–27 s; PT:11–15 s) after treatment with warfarin, suggesting that the warfarin dose was larger than expected, thus potentially increasing the risk of bleeding. Furthermore, D-dimer concentration decreased slightly in all three groups during the 12-week intervention. However, no statistically significant differences were observed among the three groups. Because D-dimer has a relatively high sensitivity in diagnosing thrombosis [22,23], these results demonstrated that the antithrombotic effects of indobufen and warfarin were similar in patients with nephrotic syndrome. Although a previous study found side effects of indobufen, including digestive complications such as nausea, diarrhea, and abdominal distension [24], our study did not observe the risk of digestive complications in patients treated with indobufen. Moreover, the warfarin group experienced more AEs during the trial, including erythema, headache, dry mouth, abdominal distension, diarrhea, and sudden cardiac death. However, given the low prevalence of AEs, no significant differences in AEs were observed among the groups.

To the best of our knowledge, our study is the first to provide evidence corroborating indobufen’s effectiveness and safety in preventing thromboembolic events in patients with nephrotic syndrome. Although no major bleeding occurred in any of the three groups, we recorded a numerically higher rate of clinically relevant minor bleeding (11/60 vs. 4/120) in the warfarin group than in the indobufen group. Moreover, this study found that treatment with warfarin requires frequent surveillance of INR and poses a higher risk of bleeding, whereas indobufen potentially offers a less burdensome treatment.

Notwithstanding, this study has certain limitations. The sample size was relatively small and the duration of follow-up was 12 weeks. Considering the median number of days to the first venous thromboembolism in patients with nephrotic syndrome was 272 days [25], the follow-up duration in our study was shorter than expected, which may be associated with unobserved thromboembolic events during follow-up. In our study, only 11 (23.9%), 11 (21.6%), and 13 (27.7%) patients’ SAlb levels remained below 28 g/L by the end of the trial in groups A, B, and C, respectively, suggesting that most patients had recovered from nephrotic syndrome after receiving treatment for three months. This indicates that the three-month intervention in this study was acceptable. A greater density of patients and longer follow-up period would be useful in defining the advantages and disadvantages of indobufen more definitively. Moreover, the use of an open-label design introduces the risk of ascertainment bias. Furthermore, 20% of the patients were lost to follow-up after 12 weeks, an occurrence predominantly caused by the COVID-19 pandemic. Finally, patients were given warfarin once daily while indobufen was given twice daily, which may introduce potential observer bias.

In conclusion, indobufen, an antiplatelet drug with an anticoagulation effect, provided equivalent effects to warfarin in preventing thromboembolic events and was associated with a reduced risk of bleeding events in adult patients with nephrotic syndrome. Therefore, indobufen is recommended for the prevention of thromboembolic events in patients with nephrotic syndrome. Nonetheless, further large-scale, randomized controlled trials should be performed to verify the efficacy and safety of indobufen in patients with nephrotic syndrome.

## Supplementary Material

Supplemental Material

## Data Availability

The datasets used and analyzed during the current study are available from the corresponding author on reasonable request.
