# Supplementary material for: Comparison of the prophylactic antithrombotic effect of indobufen and warfarin in patients with nephrotic syndrome: a randomized controlled trial
Source: Ren Fail. 2023 Jan 13;45(1):2163505. doi: 10.1080/0886022X.2022.2163505 (PMC9848225; doi:10.1080/0886022X.2022.2163505)
Supplement: Supplemental Material [file IRNF_A_2163505_SM6491.pdf]

Table S1. The changes of Hb among groups and varies visits

Effect	Dummy variable	Partial regression coefficent	Standard error	Df	t value	P value
Intercept		132.38	2.5561	177	51.79	<.0001
group	2	-1.2549	3.3545	295	-0.37	0.7086
group	3	-4.1146	3.3884	295	-1.21	0.2256
group	1	0	.	.	.	.
time	8	8.6863	1.6573	295	5.24	<.0001
time	12	7.1062	1.7171	295	4.14	<.0001
time	0	0	.	.	.	.

Table S2. The changes of PLT among groups and varies visits

Effect	Dummy variable	Partial regression coefficent	Standard error	Df	t value	P value
Intercept		232.59	7.9731	177	29.17	<.0001
group	2	11.4757	10.5808	295	1.08	0.2790
group	3	20.7080	10.6794	295	1.94	0.0534
group	1	0	.	.	.	.
time	8	-16.7562	4.7528	295	-3.53	0.0005
time	12	-18.6339	4.9272	295	-3.78	0.0002
time	0	0	.	.	.	.

Table S3. The changes of Salb among groups and varies visits

Effect	Dummy variable	Partial regression coefficent	Standard error	Df	t value	P value
Intercept		23.8712	0.8833	177	27.02	<.0001
group	2	-0.2729	1.1546	300	-0.24	0.8133
group	3	-1.0366	1.1650	300	-0.89	0.3743
group	1	0	.	.	.	.
time	8	8.6101	0.5993	300	14.37	<.0001
time	12	10.9115	0.6204	300	17.59	<.0001
time	0	0	.	.	.	.

Table S4. The changes of BUN among groups and varies visits

Effect	Dummy variable	Partial regression coefficent	Standard error	Df	t value	P value
Intercept		6.0063	0.3327	176	18.05	<.0001
group	2	0.4483	0.4311	300	1.04	0.2993
group	3	0.1710	0.4349	300	0.39	0.6944
group	1	0	.	.	.	.
time	8	0.6947	0.2279	300	3.05	0.0025
time	12	0.2670	0.2355	300	1.13	0.2579
time	0	0	.	.	.	.

Table S5. The changes of Scr among groups and varies visits

Effect	Dummy variable	Partial regression coefficent	Standard error	Df	t value	P value
Intercept		76.2588	3.6328	177	20.99	<.0001
group	2	-2.3901	4.4074	302	-0.54	0.5880
group	3	-8.6380	4.4616	302	-1.94	0.0538
group	1	0	.	.	.	.
time	8	4.7510	3.3111	302	1.43	0.1524
time	12	-1.2924	3.4120	302	-0.38	0.7051
time	0	0	.	.	.	.

Table S6. The changes of APTT among groups and varies visits

Effect	Dummy variable	Partial regression coefficent	Standard error	Df	t value	P value
Intercept		25.9139	1.0928	175	23.71	<.0001
group	2	-0.7799	1.4631	508	-0.53	0.5942
group	3	5.1984	1.4655	508	3.55	0.0004
group	1	0	.	.	.	.
time	1	-0.4737	0.8326	508	-0.57	0.5696
time	4	1.2774	0.5720	508	2.23	0.0260
time	8	-1.3016	0.5637	508	-2.31	0.0213
time	12	-1.0022	0.5926	508	-1.69	0.0914
time	0	0	.	.	.	.

Table S7. The changes of PT among groups and varies visits

Effect	Dummy variable	Partial regression coefficent	Standard error	Df	t value	P value
Intercept		10.5414	0.7627	175	13.82	<.0001
group	2	-0.5419	0.9614	513	-0.56	0.5733
group	3	7.3472	0.9644	513	7.62	<.0001
group	1	0	.	.	.	.
time	1	2.7840	0.8501	513	3.27	0.0011
time	4	4.4411	0.5789	513	7.67	<.0001
time	8	2.4967	0.5744	513	4.35	<.0001
time	12	1.8180	0.6037	513	3.01	0.0027
time	0	0	.	.	.	.

Table S8. The changes of TT among groups and varies visits

Effect	Dummy variable	Partial regression coefficent	Standard error	Df	t value	P value
Intercept		18.0759	0.4795	175	37.69	<.0001
group	2	0.6710	0.5061	495	1.33	0.1855
group	3	-0.01027	0.5116	495	-0.02	0.9840
group	1	0	.	.	.	.
time	1	0.1859	0.7667	495	0.24	0.8085
time	4	0.1590	0.5304	495	0.30	0.7645
time	8	-0.8629	0.5242	495	-1.65	0.1003
time	12	0.08398	0.5439	495	0.15	0.8774
time	0	0	.	.	.	.

Table S9. The changes of FIB among groups and varies visits

Effect	Dummy variable	Partial regression coefficent	Standard error	Df	t value	P value
Intercept		4.6328	0.1658	175	27.94	<.0001
group	2	-0.03994	0.2057	505	-0.19	0.8461
group	3	-0.08790	0.2067	505	-0.43	0.6708
group	1	0	.	.	.	.
time	1	-0.6609	0.1967	505	-3.36	0.0008
time	4	-0.7028	0.1355	505	-5.19	<.0001
time	8	-0.8866	0.1333	505	-6.65	<.0001
time	12	-0.9809	0.1395	505	-7.03	<.0001
time	0	0	.	.	.	.

Table S10. The changes of D-dimer among groups and varies visits

Effect	Dummy variable	Partial regression coefficent	Standard error	Df	t value	P value
Intercept		3712.12	322.95	175	11.49	<.0001
group	2	151.72	433.96	432	0.35	0.7268
group	3	-159.02	434.79	432	-0.37	0.7147
group	1	0	.	.	.	.
time	4	-598.77	163.95	432	-3.65	0.0003
time	8	-698.80	159.51	432	-4.38	<.0001
time	12	-1057.60	170.22	432	-6.21	<.0001
time	0	0	.	.	.	.
